# Preparation and Photovoltaic Evaluation of CuO@Zn(Al)O-Mixed Metal Oxides for Dye Sensitized Solar Cell

**DOI:** 10.3390/nano13050802

**Published:** 2023-02-22

**Authors:** Mohamed Bashir Ali Bashir, Altaf Hussain Rajpar, Ethar Yahya Salih, Emad M. Ahmed

**Affiliations:** 1 Department of Mechanical Engineering, College of Engineering, Jouf University, Sakaka 72388, Saudi Arabia; 2 College of Medical Science Technologies, The University of Mashreq, Baghdad 10021, Iraq; 3 Department of Electrical Engineering, College of Engineering, Jouf University, Sakaka 72388, Saudi Arabia

**Keywords:** CuO@Zn(Al)O, mixed metal oxide, DSSC, dye N719

## Abstract

In this manuscript, a series of dye-sensitized solar cells (DSSCs) were fabricated as a function of post-processing temperature based on mesoporous CuO@Zn(Al)O-mixed metal oxides (MMO) in conjunction with dye N719 as the main light absorber; the proposed CuO@Zn(Al)O geometry was, in turn, attained using Zn/Al-layered double hydroxide (LDH) as a precursor via combination of co-precipitation and hydrothermal techniques. In particular, the dye loading amount onto the deposited mesoporous materials was anticipated via regression equation-based UV-Vis technique analysis, which evidently demonstrated a robust correlation along with the fabricated DSSCs power conversion efficiency. In detail, of the DSSCs assembled, CuO@MMO-550 exhibited short-circuit current ($J_{SC}$) and open-circuit voltage ($V_{OC}$) of 3.42 (mA/cm^2^) and 0.67 (V) which result in significant fill factor and power conversion efficiency of 0.55% and 1.24%, respectively. This could mainly be due to the relatively high surface area of 51.27 (m^2^/g) which in turn validates considerable dye loading amount of 0.246 (mM/cm^−2^).

## 1. Introduction

Dye-sensitized solar cell (DSSC) is considered as a promising candidate for the sunlight energy conversion due to its attractive superiorities such as high conversion efficiency and cost-effectiveness; DSSC was first reported in 1991 and later enhanced through a number of reports [1,2,3,4]. However, the ultimate need for non-toxic, low production cost, considerable photoelectric conversion efficiency, and simple preparation process of semiconductor photo-anodes is of vital importance for advanced DSSC consideration [5,6]. While the photo-anode, in DSSC framework, is considered as the prominent component, a number of metal oxide geometries are being established such as nickel oxide (NiO), copper oxide (CuO), zinc oxide (ZnO), and titanium dioxide (TiO_2_) [6,7,8,9,10]. Herein, mixed metal oxide (MMOs) using layered double hydroxides (LDHs) as the starting material has demonstrated superior photo-anode performance for DSSC application due to their forthright preparation process and relatively low-cost [11]. LDHs are layered anionic clays with two-dimension structure of binary and/or trinary metallic ions. The addressed structure has revealed a number of thought-provoking features through which variety of applications are proposed, especially in the field of optoelectronics [12,13]. Interestingly, thermal treatment of LDHs leads to the collapse of 2D layer structure and subsequently MMOs formation [14]. MMOs have attracted increasing attention of research societies for variety of applications such as photo-catalysts in visible and ultraviolet regions, as super-capacitors, and as anode materials [15,16,17,18]. In the field of optoelectronics, DSSCs in particular, MMOs are utilized as a potential photo-anode material/s because of their noticeably high specific surface area, similar optical band gap to that of ZnO as well as TiO_2_, fast photo-responsive performance, tunable composition, and relatively high electron-injection behavior [11,19].

To date, a number of reports have revealed the use of Zn(Al)O-MMO in DSSCs application using nanostructured Zn/Al-LDH as precursor; these reports emphasize on the crucial role of ZnO within the Zn(Al)O-MMO matrix [20,21,22,23,24,25,26,27,28,29]. ZnO with a bandgap of 3.37 eV, high-electron life, and mobility (≥10 s and 200–300 cm^2^ V^−1^ s^−1^) is well-though-out as an alternative photo-anode to that of TiO_2_ [30,31]. Due to the relatively low specific surface area of ZnO and thus a reduced dye loading amount, the above-mentioned semiconductor requires an enhancement in the addressed matter. Therefore, this study demonstrates a novel pathway for the synthesis and characterization of CuO@Zn(Al)O-MMO using CuO@Zn/Al-LDH as a precursor, and subsequently the fabrication of comparatively efficient DSSC. It was found that CuO addition to the MMO matrix significantly increased the surface area, and hence higher loading volume of dye onto the surface of the deposited semiconductor film was attained. This in turn resulted in higher photovoltaic performance as compared to that of pure Zn(Al)O-MMO.

## 2. Materials and Methods

### 2.1. Synthesis of CuO and CuO@Zn(Al)O-MMO

CuO was synthesized utilizing the conventional pulse laser ablation in water. In particular, a laser energy of 39 mJ/pulse and second harmonic wavelength of 532 nm was vertically positioned on Cu target (99.9%) in 25 mL deionized water at room temperature (RT). A continuous stirring of 350 rpm was applied during three repetitive laser ablation periods (30, 40, and 50 min). The recurrence frequency was maintained at 6 Hz with 10 ns pulse interval.

Subsequently, mesoporous CuO@Zn(Al)O-MMO using CuO@Zn/Al-LDH as a precursor was synthesized via a combination of co-precipitation approach and thermal treatment technique. In a typical synthesis process, Zn/Al-LDH as a precursor was synthesized with molar ratio of 8:1 (Zn(NO_3_)_2_.6H_2_O and Al(NO_3_)_2_.9H_2_O) in 200 mL in deionized water at RT under a stirring rate of 700 rpm. Herein, the prepared CuO solution was added to the mixture, while a homogenous crystal growth was sustained through a drop wise addition of 1.5 M NaOH to the resultant CuO@Zn/Al-LDH solution during the experiment (pH of 7). The attained light-greenish slurry was sustained at 70 °C for 12 h in an autoclave, to confirm a wide-ranging mesoporous growth. Continuously, the resultant product was subject to multi-cycled washing and centrifuging process; after which it was dried at 75 °C. The consequent paste was later deposited on fluorine tin oxide (FTO) glass using doctor blade technique (1 cm^2^). Hereinafter, CuO@Zn(Al)O-MMO was attained via thermal treatment method wherein different treatment temperatures (T = 350, 450, 550, and 650) were applied in muffle furnace for 60 min. The fabricated films/devices were designated as CuO@MMO-T, where T signifies the utilized treatment temperature.

### 2.2. DSSC Fabrication

The counter electrode (Pt) was attained on the utilized FTO glass via DC sputtering approach. Synchronously, the fabricated MMO-550 and CuO@MMO-T films were immersed in 5 mM of dye solution (Ruthenizer 535-bisTBA, N719) for 4 h. Next, the fabricated counter and photo electrodes were assembled on both sides of the polymer film (100 µm) wherein a separator and sealing elements were accomplished. Finally, Iodolyte Z50 redox couple electrolyte (iodide/triiodide) was injected between the sandwiched counter and photo electrodes via capillarity.

### 2.3. Characterization

The structural characteristics was carried out using X-ray diffraction technique (Bruker, AXS D8) under 40 kV, CuKα. radiation, and a wavelength of 1.54 nm, while the thermal analysis of pristine CuO@Zn/Al-LDH was recorded via thermogravimetric and differential thermal technique (TGA/DTG, Mettler Toledo-SBTA851). The effective thickness of the fabricated CuO@MMO-T films was measured via a profilometer P10-TENCOR (≈10 μm). The optical absorbance phenomena were evaluated using UV-Vis spectroscopy technique (Shimadzu, UV-3600). Further, the BET surface area values of the CuO@MMO-T were calculated through N2 adsorption/desorption approach (ASAP 2020, Micromeritics). The solar cell performance (I-V characteristics) was evaluated using source measure unite (Keithley 237 SMU, Cleveland, OH, USA) and light source with intensity of 100 mW·cm^−2^ in conjunction with Am 1.5 G sunlight stimulator.

## 3. Results

**Figure 1:**
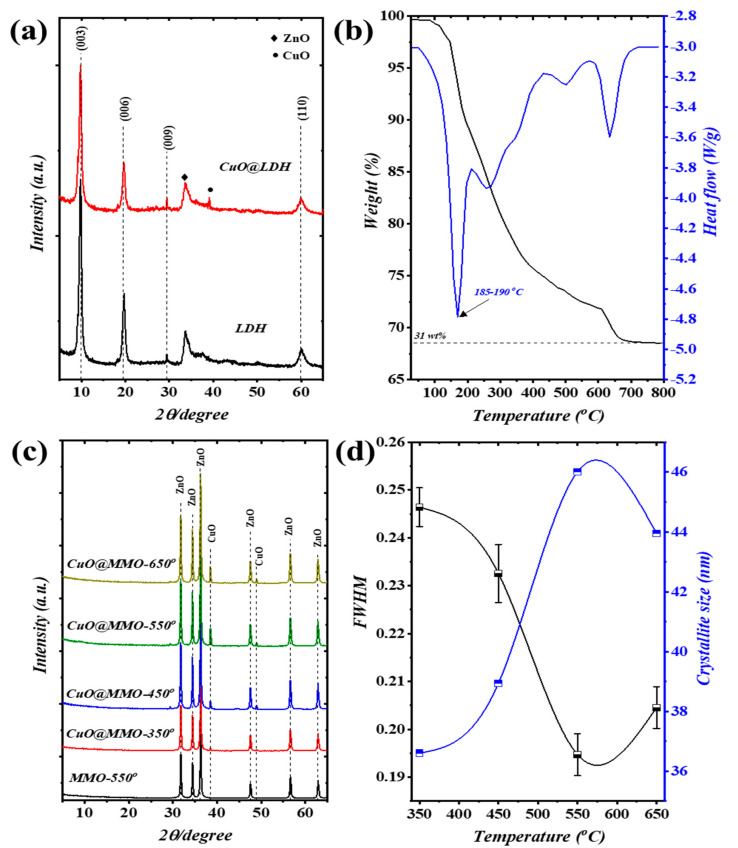
(**a**) XRD patterns of LDH and CuO@LDH, (**b**) TGA/DTG results of CuO@LDH, (**c**) XRD patterns, and (**d**) FWHM and crystallite size of the CuO@MMO-T films.

The XRD patterns of pristine CuO@Zn/Al-LDH and Zn/Al-LDH are illustrated in Figure 1a, wherein four main peaks were perceived; these systematically correspond to specific planes (003), (006), (009), and (110). The addressed observation evidences the attainment of LDH crystal orientation (JCPDS-No: 38–0486) [32]. Furthermore, additional peaks obtained at around $2\theta \;34.6^{{}^{\circ}}$ and $48.2^{{}^{\circ}}$ are, respectively, indexed to the crystal formation of ZnO and CuO phases [33,34]. Figure 1b depicts the thermal analysis of CuO@Zn/Al-LDH using TGA/DTG approach. Three distinguishable stages of weight loss were attained in the TGA profile showing Δm≈31% degradation attitude. In particular, weight loss occurring up to $200{\;}^{{}^{\circ}}C$ is attributed to the moisture evaporation ($\Delta m_{1}\approx 10\%$) and release of the bound water; this particular observation is verified with the obtained peak on DTG profile at $185–190{\;}^{{}^{\circ}}C$. Spontaneously, the dominant decomposition process of LDH brucite-like layers is covered within the range from $200–500{\;}^{{}^{\circ}}C$ along with H_2_O and strong CO_2_ release ($\Delta m_{2}\approx 15\%$). The thermal decomposition process acquired at $500{\;}^{{}^{\circ}}C$ and above have relatively low weight loss value ($\Delta m_{1}\approx 5\%$) which in turn can be attributed to the recrystallization of ZnO and CuO phases [35]. The DTG profile revealed similar outcomes as those observed with the DTG findings. In conjunction with the TGA/DTG findings, Figure 1c elucidates the XRD patterns of MMO-550 as well as CuO@MMO-T films by means of post-processing temperature. The presented XRD patterns confirm phase alteration of pristine LDH to polycrystalline MMOs subsequent to treatment temperature, collapse of 2D-LDH structure. Particularly, two crystal formations were attained which are individually indexed to hexagonal structure of ZnO (PDF: 89–1397) as well as CuO formation (PDF: 48–1548). It is a well-established fact that the FWHM is a crystal quality indicator in accordance with Debye framework [36]. Hence, increasing the post-processing temperature from $350{\;}^{{}^{\circ}}C$ to $550{\;}^{{}^{\circ}}C$ resulted in higher crystal quality of CuO phase at $48.6^{{}^{\circ}}$ (Figure 1d). The crystallite size of the prepared samples was estimated in accordance with Debye–Scherrer equation as follows [37]:

(1) $$D=\frac{K\lambda }{\beta \;\cos\theta }$$

herein, the shape factor is represented by *K*, *λ* is the utilized wavelength during XRD test, *β* is the full width at half maximum, and *θ* is the Bragg angle of diffraction.

**Figure 2:**
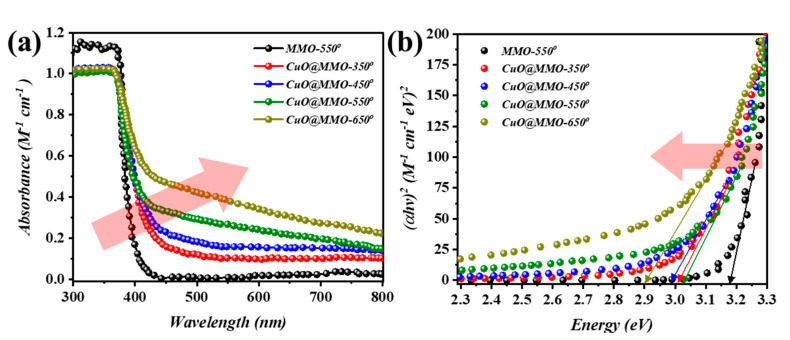
(**a**) Ultraviolet-visible light absorption spectra and (**b**) evaluated band gap of annealed films.

Figure 2a demonstrates the UV-Vis absorption spectra of the deposited films. The cut-off phenomenon for MMO-550 film at around 380 nm is mainly due to the ZnO. However, a slight Batho-chromic shift was noticed after CuO addition to the MMO matrix; this particular observation can be clearly noticed at higher thermal treatment temperature. This in turn was also supported through the optical band gap determination (Figure 2b) using Tauc relation [38]. In detail, MMO-550 exhibited an optical band gap of 3.2 eV after which a band gap of 3.03 eV was acquired for CuO@MMO-350. In the meanwhile, CuO@MMO-650 demonstrated band gap of 2.9 eV.

The isotherms of N_2_ adsorption/desorption analysis for the deposited films, MMO-550 and CuO@MMO-T, revealed the occurrence of H3 hysteresis loop, Type IV, according to IUPAC (Figure S1 from the Supplementary Materials) [39]. Instantaneously, the pore width distribution of the deposited films evidences the occurrence of mesoporous structure oriented within the range of 10–90 nm [40], Figure S2. The evaluated BET surface area is presented in Figure 4d, through which it can be concluded that higher thermal treatment resulted in higher value of the specific surface area.

**Figure 3:**
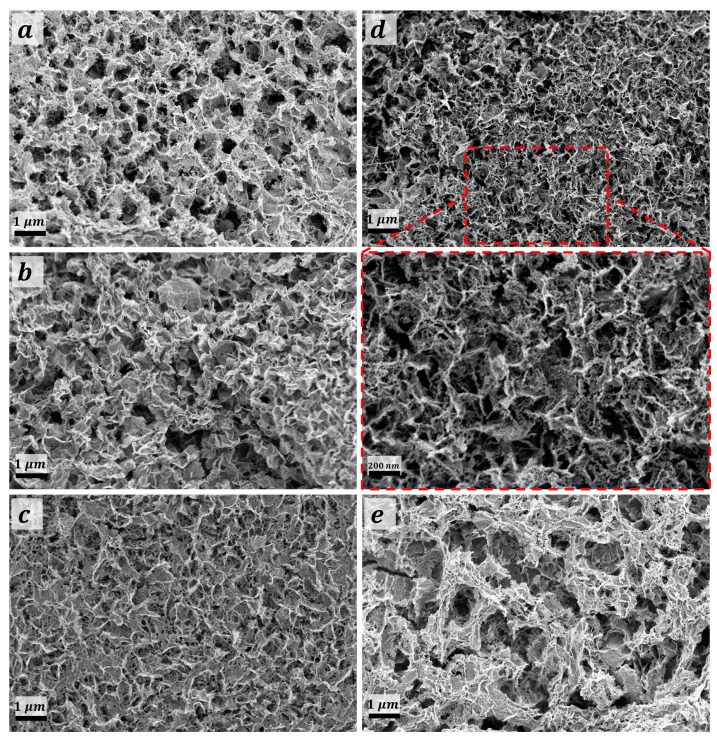
FE-SEM topographies of (**a**) MMO-550°, and CuO@MMO-T treated at (**b**) 350°, (**c**) 450°, (**d**) 550°, and (**e**) 650°.

Figure 3a–e shows the FE-SEM topographies analysis of the deposited films with respect to the thermal treatment temperature. MMO-550 film (Figure 3a) revealed the occurrence of mesoporous non-uniform sheet-like morphology. The addressed sheet-like morphology was found to be perpendicularly oriented on the FTO substrate for CuO@MMO-350 film which could be due to the relatively low thermal treatment temperate; this particular observation was not noticed with thermal treatment at 450 °C, Figure 3c. Thermal treatment at 550 °C resulted in an upright orientation sheet-like morphology with mesoporous nature. However, higher temperature, 650 °C, revealed the distortion of the aforementioned morphology which decreased the subsequent dye loading amount and/or surface area value. The average sheet-thickness was evaluated to be 47 nm, 41 nm, 36 nm, and 51 nm for films treated at 350 °C, 450 °C, 550 °C, and 350 °C, respectively; the average sheet thickness for CuO@MMO-T was found to be 43.75 nm. This could demonstrate an active role in higher dye loading amount as a function of the sheet thickness. Similarly, the average surface porosity of the deposited materials was found to be 129 nm, 136 nm, 134 nm, and 115 nm, respectively. The latter was observed to be, to a certain extent, in a good agreement with the attained results using the pore width distribution, Figure S2.

**Figure 4:**
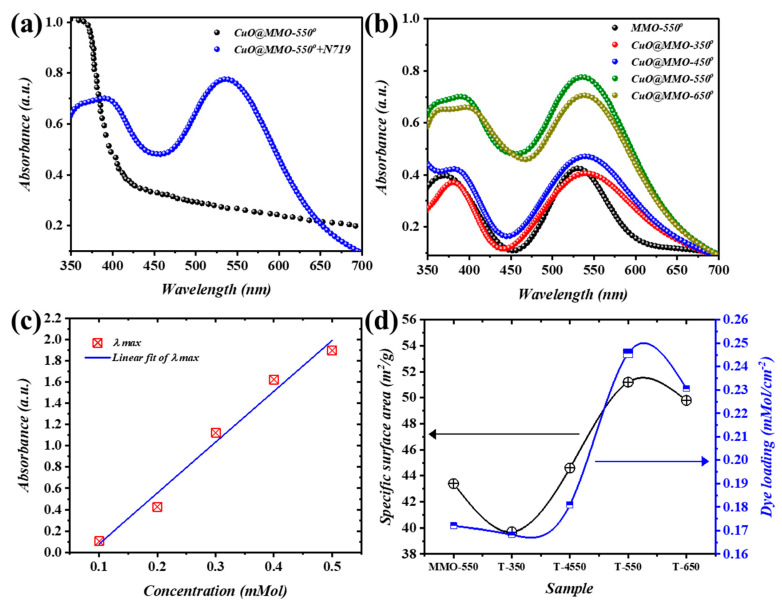
(**a**) Absorption spectra of MMO-550° and CuO@MMO-550° containing N719, (**b**) spectral response of the annealed films, (**c**) linear fit of maximum absorbance @ 525 nm, and (**d**) variation of the specific surface area (black) and dye N719 loading amount (blue).

Figure 4a demonstrates the absorbance outcomes of CuO@MMO-550 together with CuO@MMO-550 containing N719 as a sensitizer. A cut-off absorption wavelength is centered within the range of 380 to 410 nm of CuO@MMO-550, whereas another obvious cut-off wavelength at approximately 550 nm is mainly induced via the dye molecules [41]. All thermally treated films contributed similar trends (Figure 4b) at different intensities; thus, the N719 loading onto the deposited films’ surfaces is directly proportional to the absorption peak at 550 nm. Henceforth, the BET surface area trend can be validated through the estimation of N719 loading amount using UV-Vis results analysis. The absorption peaks at five continuous concentrations of N719 are elucidated in Figure 4c. Jointly, a fitting curve was produced using the addressed absorption peak after which the attained equation (y=4.777x−0.399) was utilized for the amount of N719 loaded estimation. It was proven, therefore, that an augment in the post-treatment temperature resulted in higher N719 loading wherein CuO@MMO-550 exhibited the highest loading value (0.246 mMol/cm^2^). The obtained N719 loading amount trend was anticipated from the BET surface area results (Figure 4d) which evidently supports the proposition that mesoporous profile of CuO@MMO-T allows molecules transportation within an organized mesoporous geometry. This in turn can validate higher light harvesting in an organic framework.

**Figure 5:**
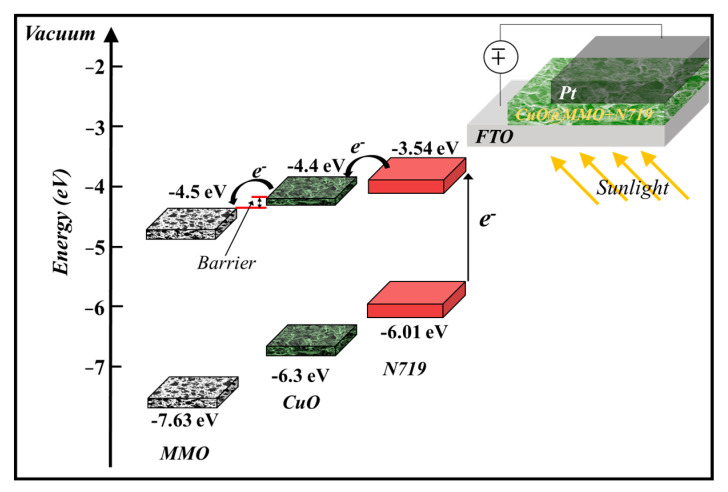
The proposed geometry band diagram [29,43,44].

The proposed geometry energy band illustration (Figure 5) of the deposited CuO@MMO-T alongside the employed N719 suggests that photons of light are absorbed through the molecules of dye n719 wherein electrons are excited from HOMO to LUMO. Subsequently, the obtained electrons are injected within the CuO@MMO matrix resulting in current flow in the outer circuit [42].

**Figure 6:**
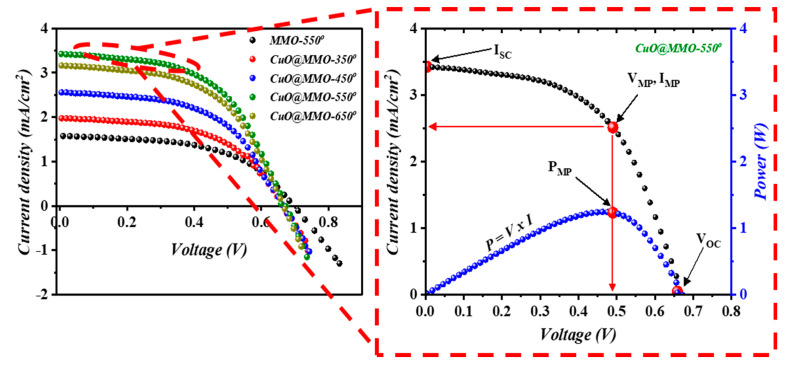
I-V characteristics of the DSSC devices; inset is the current density and power variation as a function of voltage.

Figure 6 shows the J−V characteristics of the fabricated DSSC devices; inset into Figure 6 illustrates a variation of current density and power versus the applied voltage for the optimum device (CuO@MMO-550°). DSSC fabricated with thermal treatment temperature of 550° demonstrated the highest short circuit current density ($J_{SC}=3.42\;\mathrm{mA}/{\mathrm{cm}}^{2}$), while all DSSC devices revealed almost similar open circuit voltage ($V_{OC}\approx 0.66$). Correspondingly, the optimum DSSC exhibited a power conversion efficiency (PCE) of 1.24%; this was found to be 110.2% higher than MMO-550° DSSC (0.59%) which outlines the important character of CuO in the MMO geometry. The results suggest that an introduction of CuO within MMO matrix may allow higher rate of electron transition through the DSSC device [45], which could be due to an enhanced surface properties which in turn led to higher dye N719 loaded onto the deposited CuO@MMO surface, as clearly can be noticed from Table 1. The J−V curves demonstrated similar trend as that obtained in the BET and dye loading amount profiles, Figure 4d, which signposts a favoring light photon harvesting because of an enhanced surface area as well as the associated higher dye loading in the proposed mesoporous geometry. It should be mentioned that the attained relatively low fill factor is mainly due to charge transfer resistance within the counter electrode [46,47].

Table 1 tabulates an in-depth photovoltaic parameters of the fabricated DSSC devices. In the meanwhile, Table 2 represents the proposed study optimum J-V characteristics (CuO@MMO-550°) in comparison to the reported studies utilizing MMO-based Zn/Al-LDH as the anode material for DSSC application.

**Figure 7:**
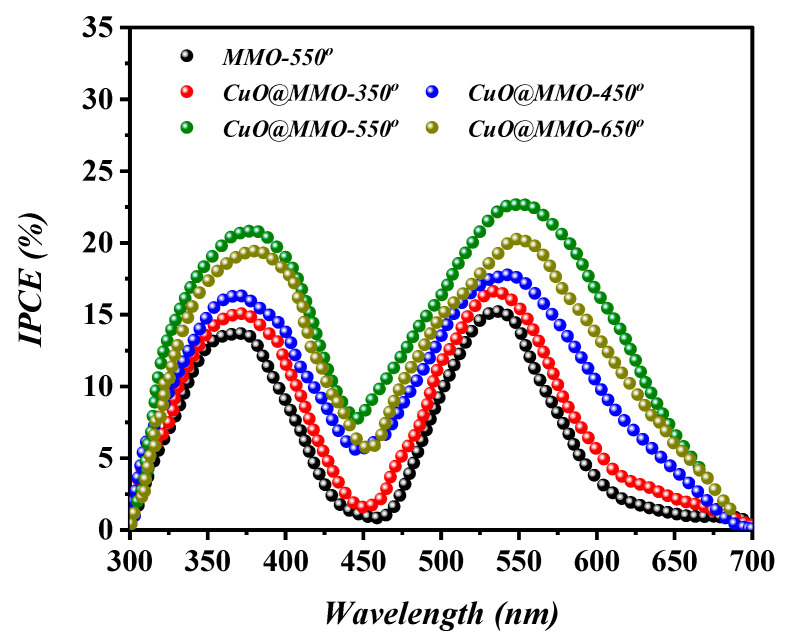
IPCE curves of the fabricated DSSC devices.

Figure 7 elucidates the IPCE curves based on MMO-550° and CuO@MMO-T DSSC devices, where two obvious peaks can be perceived at around 380 nm and above 550 nm. The former, in the UV region, is mainly resulted from the self-excitation of MMO semiconductor, while the latter is due to the light absorption via dye N719 sensitizer. Additionally, the IPCE profile demonstrated similar behavior to that attained in the J−V characteristics analysis (Figure 6); this indicates higher current density at higher photons to electrons conversion proportion. The low intensity phenomenon of IPCE curves within the range of 400–500 nm suggests low light consumption resulting in deteriorated performance of the fabricated DSSC.

## 4. Conclusions

Continuous mesoporous CuO@Zn(Al)O-MMO films were successfully deposited on FTO glass substrate for DSSC application using Zn/Al-LDH as precursor. Subsequently, the DSSC devices were fabricated as a function of thermal treatment temperature. The amount of dye N719 loaded onto the deposited films was estimated using a regression equation through which an evidenced correlation with the power conversion efficiency of the DSSC devices was established. Specifically, the optimum sample, CuO@MMO-550, with surface area of 51.27 (m^2^/g) and loaded dye amount of 0.246 (mM/cm^−2^) revealed the occurrence of $J_{SC}$ and $V_{OC}$ of 3.42 (mA/cm^2^) and 0.67 (V), respectively. Concurrently, the addressed device demonstrated FF and PCE of 0.55% and 1.24%, respectively.

## Figures and Tables

**Table 1:** In-depth surface and photovoltaic parameters of the fabricated DCCS devices.

Sample	Sheet-Thickness (nm)	BET (m^2^/g)	Dye (mM/cm^−2^)	J_sc_ (mA/cm^2^)	V_oc_ (V)	FF (%)	PCE (%)
MMO-550°	45	43.42	0.172	1.57	0.68	0.55	0.59
CuO@MMO-350°	47	34.74	0.168	1.97	0.65	0.54	0.71
CuO@MMO-450°	41	44.61	0.181	2.56	0.66	0.54	0.92
CuO@MMO-550°	36	51.27	0.246	3.42	0.67	0.55	1.24
CuO@MMO-650°	51	49.80	0.231	3.16	0.66	0.55	1.14

**Table 2:** Photovoltaic characteristics of the current work as compared to reported studies.

Materials	Jsc (mAcm^−2^)	Voc (V)	FF (%)	PCE (%)	Ref.
Zn/Al-LDH	0.073	0.430	0.416	0.0129	[11]
Zn/Al-LDH	0.43	2088	0.44	0.55	[21]
Zn/Al-LDH	2.03	0.69	0.72	1.02	[42]
Zn/Al-LDH	1.47	0.64	0.73	0.69	[29]
Zn/Ti-LDH	0.54	5.32	0.54	1.57	[23]
GO@Zn/Al-LDH	0.37	4.46	0.34	0.44	[26]
G@Zn/Al-LDH	0.36	3.62	0.39	0.51	[25]
TiO2@Zn/Al-LDH	2.63	0.81	0.70	1.50	[20]
CuO@Zn/Al-LDH	3.42	0.67	0.55	1.24	This study

## Data Availability

The data demonstrated in the proposed study are accessible upon reasonable request.

## Supplementary Materials

The following supporting information can be downloaded at: https://www.mdpi.com/article/10.3390/nano13050802/s1. Figure S1: Nitrogen gas adsorption/desorption isotherms of annealed films of the annealed films. Figure S2: Pore diameter distribution of MM-550 and CuO@MMO-T.
