# Supplementary material for: Preparation and Photovoltaic Evaluation of CuO@Zn(Al)O-Mixed Metal Oxides for Dye Sensitized Solar Cell
Source: Nanomaterials (Basel). 2023 Feb 22;13(5):802. doi: 10.3390/nano13050802 (PMC10005446; doi:10.3390/nano13050802)
Supplement: Supplementary file 1 [file nanomaterials-13-00802-s001.zip › nanomaterials-2202668-supplementary.pdf]

nanomaterials

Supplementary Materials

Preparation and Photovoltaic Evaluation of CuO@Zn(Al)O-Mixed Metal Oxides for Dye Sensitized Solar Cell

Mohamed Bashir Ali Bashir [1,*], Altaf Hussain Rajpar [1], Ethar Yahya Salih [2] and Emad M. Ahmed [3]

[1] Department of Mechanical Engineering, College of Engineering, Jouf University, 72388 Sakaka, Saudi Arabia; ahrajpar@ju.edu.sa
[2] College of Medical Science Technologies, The University of Mashreq, 10021 Baghdad, Iraq; ethar988@gmail.com
[3] Department of Electrical Engineering, College of Engineering, Jouf University, 72388 Sakaka, Saudi Arabia; emamahmoud@ju.edu.sa
* Correspondence: mbashir@ju.edu.sa

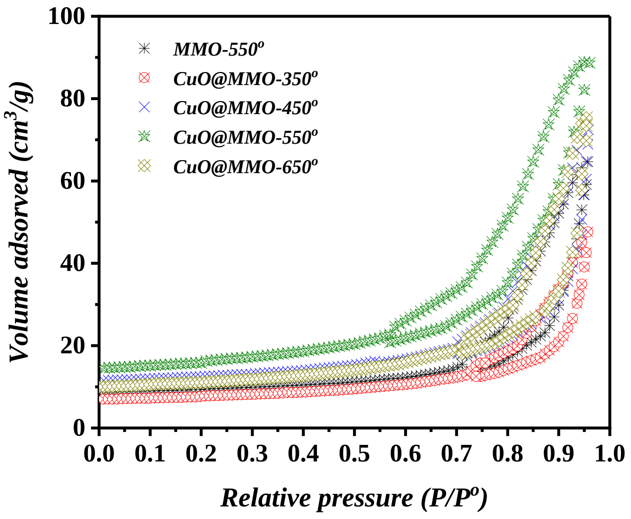

Figure S1. Nitrogen gas adsorption/desorption isotherms of annealed films of the annealed films.

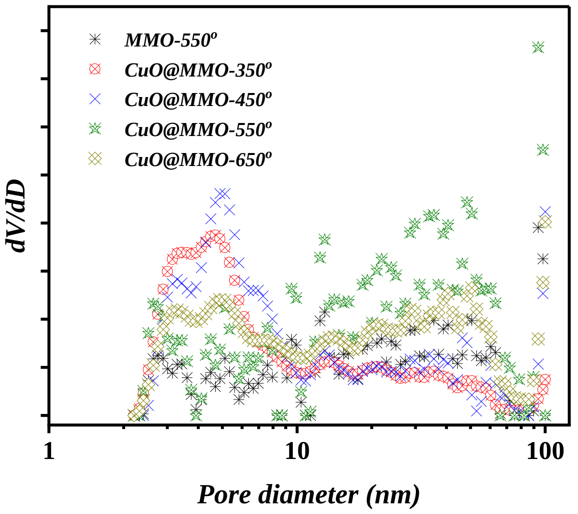

Figure S2. Pore diameter distribution of MM-550 and CuO@MMO-T.
